# Reflections on Australian radiology education: How can we innovate and improve?

**DOI:** 10.1111/1754-9485.13786

**Published:** 2024-10-18

**Authors:** Sally L Ayesa

**Affiliations:** ^1^ Faculty of Medicine and Health University of Sydney Camperdown New South Wales Australia; ^2^ Department of Nuclear Medicine Royal North Shore Hospital St Leonards New South Wales Australia

**Keywords:** education, medical education, radiology

The annual Association of Academic Radiology (AAR) Conference celebrates the art of medical imaging education, bringing together hundreds of like‐minded radiologists, mostly from North American universities and radiology residency programs. When I first attended in 2023, I was struck by the stark contrast between the structure of medical school and specialist education between the United States and Australia. Radiology training programs in the United States are directly linked with universities rather than local public health networks as they are in Australia. The inherent connection between these universities and their affiliated academic radiologists provides fertile ground for radiology education to be spiralled through medical school into post‐graduate education.

As an international delegate, my attendance prompted reflection on what the Australian imaging community could learn from the AAR, noting published and anecdotal concerns regarding radiology's engagement with medical schools and other non‐radiologist clinical learners.^1^, ^2^, ^3^ In addition, the recent implementation of the new programmatic assessment‐based training program for Royal Australian and New Zealand College of Radiologists (RANZCR) trainees has meant that there is an increased onus on local Clinical Supervisors and Directors of Training to provide high‐level educational experiences and resources.^4^

Considering our local zeitgeist and what I have learned from our North American colleagues, I present my thoughts on enhancing education for our medical students, junior doctors and training registrars.

## Radiologists should dedicate more time and resources to learning how to teach

Radiologists are often expected to act as expert teachers even though they have undergone no formal training in how to achieve this. We assume that our trainees will gain this skill through the osmosis of radiology training and self‐directed learning, and too often glancing over the nuances of effective teaching. There is, however, specific knowledge surrounding successful connection with audiences of different skill levels and educational needs, and the fundamentals of designing effective demonstration aids for different teaching contexts. Recently, the importance of medical education as a professional skill has been formalised within the ‘Intrinsic Roles’ section of the RANZCR Curriculum.^5^ With this, RANZCR as an organisation, as well as local training sites, will need to reflect on how we can impart this core knowledge to our trainees in a meaningful way.

As radiology education sessions increasingly migrate to hybrid or wholly online formats, we also need to consider how our teaching needs to adapt to reach an audience separated in space and often time. In my personal experience working with online content delivery, I can see that even some of the most accomplished medical educators have difficulty connecting with audiences online – even though they can enthral a room when presenting in person. Conveying one's love and passion for the subject matter through the screen to the too‐often faceless and often unresponsive audience is easier said than done.

## Medical imaging educators need to adjust their styles to reach younger generations of learners

Research and prevailing opinion indicate that Millennial and post‐Millennial (Generation Z) learners have different expectations and approaches to learning radiology, including the integration of digital resources, a desire for more collaborative and flexible learning environments, and a focus on educational activities where students gain a personal sense of value and meaning.^6^ As an older Millennial, I have to consistently adjust content creation to engage the current generations of students and radiologists‐in‐training.

Social media and online learning environments are playing a greater role in learning, which can be less familiar territory for older generations of educators. The COVID‐19 pandemic accelerated the revolution in online radiology education with the now widespread integration of live online and pre‐recorded educational sessions. Delivery options are now flexible, breaking down the geographic and time barriers to accessing quality content and inherently more suitable to the natural learning styles of younger learners. Considering challenges, millennial and post‐millennial learners are noted to have shorter attention spans and a greater desire for active engagement and/or demonstration in digital classrooms, placing the burden on the radiologist educator to deliver within these parameters.^7^

## There should be a national medical imaging curriculum for Australian (and New Zealand) medical schools driven by RANZCR

One of the greatest challenges I face as an academic radiologist is ensuring our content matches the medical imaging competence we expect an intern to possess on their first day of practice. While I have an estimate of this benchmark based on my own clinical and educational experience, I lack the backing of an agreed educational standard for graduating Australian doctors. Curriculum documents exist in Europe, the United Kingdom and North America arising from consensus agreements from groups such as the European Society of Radiology, the Royal College of Radiologists, AAR and AMSER (Alliance of Medical Student Educators in Radiology).^8^, ^9^, ^10^

These documents exist to ensure that there is an agreed minimum standard of educational delivery for medical schools and to guide local academics and clinical educators regarding the nature and complexity of knowledge and skills they need to share. With RANZCR's formalisation of professional skills competencies alongside radiodiagnosis and procedural knowledge, it should be our prerogative as radiologists to shape the narrative of our specialty. This includes modelling how it is integrated into clinical practice and showcased as a viable and exciting subspecialty option for medical students.

## Australian and New Zealand medical educators should have their own network

From my own experience, I know radiologists often face challenges in breaking down professional silos and integrating education with practice. Many of us love to teach but struggle to find opportunities to work with students and/or balance teaching against clinical and personal commitments. Those of us who do teach can find ourselves disconnected from the broader like‐minded community of imaging educators.

In the year since attending my first AAR Conference, I have noticed small changes in my teaching practice, including my approach to designing educational material and engaging learners. Specific innovations range from small adjustments, such as including a ‘toolbox’ slide of arrows and annotations in my PowerPoint presentations, to larger reflections and research projects examining the relationships between Australian universities and the medical imaging community.

This conversation needs to consider both what the radiology community can do for our learners and what the institutions can do for us to add value to the teaching experience. Establishing a network similar to AAR could connect and motivate us to engage more readily with teaching opportunities. With education skills now considered examinable for RANZCR trainees, there is also the opportunity to dedicate more resources to radiology education as a stand‐alone discipline.

In conclusion, Australia's medical imaging education community consists of a dedicated and diverse group of professionals with boundless experience to share with future generations of doctors. As a recently qualified specialist, I feel proud to be part of this community and grateful to the mentors and role models who have shaped me professionally. I present my reflections not as a criticism but as a prompt to consider how we can grow and adapt as the educational paradigm shifts. As radiologists, we should be driving the perception of our specialty and us ourselves setting the parameters for how radiology is taught in universities and hospitals.

## Data Availability

Data sharing not applicable to this article as no datasets were generated or analysed during the current study.
